# Pedophilic sex offender show reduced actiation in the right dlpfc during integration of emotion and cognition – preliminary results

**DOI:** 10.1192/j.eurpsy.2021.1102

**Published:** 2021-08-13

**Authors:** J. Szczypiński, M. Wypych, A. Michalska, A. Krasowska, M. Kopera, H. Suszek, A. Marchewka, A. Jakubczyk, M. Wojnar

**Affiliations:** 1 Department Of Psychiatry, Medical University of Warsaw, Warszawa, Poland; 2 Laboratory Of Brain Imaging, Nencki Institute of Experimental Biology, Warszawa, Poland; 3 Faculty Of Psychology, University of Warsaw, Warszawa, Poland

**Keywords:** Pedophilia, child sexual offending, emotion, cognition

## Abstract

**Introduction:**

The pedophilic disorder is characterized by a sexual preference for children and leads to child sexual abuse (CSA) in half of the patients. Studies showed that pedophiles with a history of CSA (CSA+) are inferior, in inhibitory control, to those without (CSA-).

**Objectives:**

Inhibitory control may be influenced by negative affectivity, which was shown to be a state factor facilitating sexual abuse. Nevertheless, it is not known if distress influence CSA+ and CSA- equally.

**Methods:**

We recruited three groups of participants: healthy controls (HC) CSA+ and CSA- who performed an emotional Go-NoGo block task. The task was design specifically to correspond to a situation in which an indivisual is opposed by a negative life event. In each trial, participants were presented with photographs, either of neutral or negative valence, which did not require reaction. After the photographs, a circle (Go stimuli) or a square (NoGo stimuli) was presented.

**Figure:**
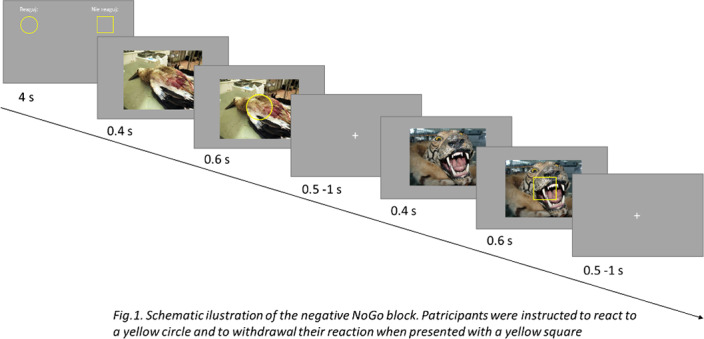

**Results:**

We found that HC and CSA- had slower reaction time in negative compared to neutral condition (regardless of the block type), while CSA+ did not. Consequently, HC and CSA- showed increased activation in the right dorsolateral prefrontal cortex (DLPFC) in negative compared to the neutral condition, what was not observed in CSA+.

**Figure:**
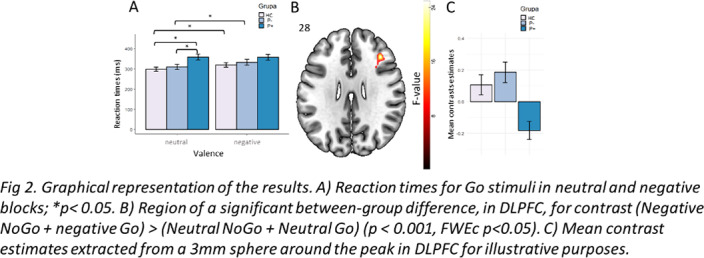

**Conclusions:**

DLPFC is crucial for cognitive control, however, the activity of this region is modulated by emotional valence. Reduced engagement of dlPFC in CSA+ in negative condition (irrespectively of the task instructions), suggest that negative emotions in CSA+ disrupt also other aspects of cognitive control, rather than inhibition specifically.

